# Sentinel interaction mapping – a generic approach for the functional analysis of human disease gene variants using yeast

**DOI:** 10.1242/dmm.044560

**Published:** 2020-07-08

**Authors:** Barry P. Young, Kathryn L. Post, Jesse T. Chao, Fabian Meili, Kurt Haas, Christopher Loewen

**Affiliations:** Department of Cellular and Physiological Sciences, Life Sciences Institute, University of British Columbia, Vancouver, BC V6T 1Z3, Canada

**Keywords:** Yeast, Variants, Human disease genes

## Abstract

Advances in sequencing technology have led to an explosion in the number of known genetic variants of human genes. A major challenge is to now determine which of these variants contribute to diseases as a result of their effect on gene function. Here, we describe a generic approach using the yeast *Saccharomyces cerevisiae* to quickly develop gene-specific *in vivo* assays that can be used to quantify the level of function of a genetic variant. Using synthetic dosage lethality screening, ‘sentinel’ yeast strains are identified that are sensitive to overexpression of a human disease gene. Variants of the gene can then be functionalized in a high-throughput fashion through simple growth assays using solid or liquid media. Sentinel interaction mapping (SIM) has the potential to create functional assays for the large majority of human disease genes that do not have a yeast orthologue. Using the tumour suppressor gene *PTEN* as an example, we show that SIM assays can provide a fast and economical means to screen a large number of genetic variants.

## Abstract (precis)

**Summary:** Sentinel interaction mapping is a technique that employs systematic genetic interaction mapping to identify yeast-human genetic interactions, which can be used to rapidly functionalize human disease genes and their variants.

## INTRODUCTION

The most recent version of the Human Gene Mutation Database identifies 203,885 disease-associated mutations (Stenson et al., 2017). Validating and verifying the nature of these genetic variants represents a formidable yet vitally important task in understanding the genetics of human disease. Many different approaches are being developed to untangle this data, including *in silico*, *in vitro* and *in vivo* techniques. The budding yeast *Saccharomyces cerevisiae* has been shown to be a useful tool in understanding mammalian cellular processes, combining the ease of manipulation of a simple unicellular organism with the conservation of fundamental biological processes found in all eukaryotes (Botstein and Fink, 2011).

**Fig. 1.**
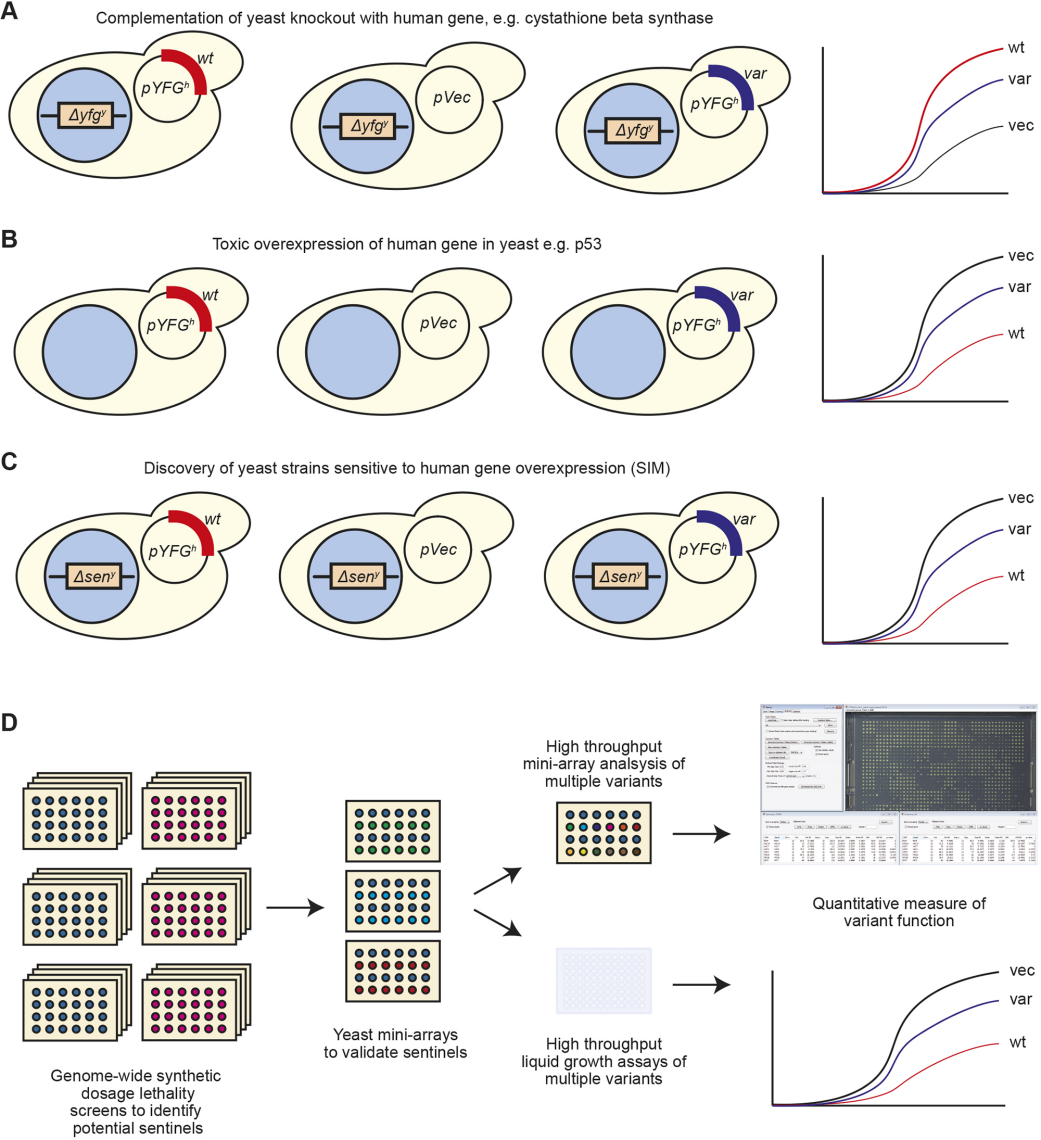
**Functionalization of human disease gene variants in yeast.** (A) Complementation of the deletion of a yeast gene (*YFG^y^*) with its human orthologue (*YFG^h^*). (B) Overexpression of a human gene, resulting in toxicity in yeast. (C) Overexpression of a human disease gene in a specifically sensitive yeast deletion strain (‘sentinel’ - *Δsen^y^*). (D) Overview of SIM. Synthetic dosage lethality screening used to identify candidate strains. These strains are then validated in mini-array format with a limited number of gene variants to identify useful sentinels. Sentinels are then employed in high-throughput mini-array or liquid growth assays to functionalize variants.

Analysis of human gene variants in yeast can be achieved through a variety of techniques. If the gene has a yeast orthologue, then the ability of the human gene to complement this deletion can be used (Sun et al., 2016), an early example of this being the human gene cystathioine beta-synthase (Kruger and Cox, 1995). Such a system requires two criteria to be met. First, the deletion of the yeast gene must confer a phenotype (usually growth related) that can be assayed. Second, the human gene must be able to successfully reverse this phenotype. Variants of the gene may then be assayed by the degree to which they achieve this (Fig. 1A), the assumption being that non-functional variants will fail to rescue growth. This approach has also been extended to paralogous genes (Yang et al., 2017). However, a possible drawback to this technique is that rescue by an orthologue may only reflect the complementation of a specific function of the protein, namely that which leads to a growth defect. For a protein with multiple functions, simple complementation testing will not be able to report on those activities that do not lead to a growth phenotype. Although additional activities can be reported on by using a specific reporter assay, such as the yeast transcription reporting of aggregating proteins technique (Newby et al., 2017), such systems require a detailed knowledge of the underlying biology of the gene in question in order to develop these assays.

In cases where there is no yeast orthologue of the gene of interest, overexpression of the human gene may lead to a growth phenotype in wild-type (wt) yeast (Fig. 1B). One study estimates that ∼5% of human genes, when overexpressed in yeast, will repress growth (Sekigawa et al., 2010). The tumour suppressor gene p53 (*TP53*) is a well-characterized example, where a growth defect is seen in yeast upon overexpression of the wt gene, but not with loss-of-function (LoF) variants (Hadj Amor et al., 2008).

However, in cases where there is no yeast orthologue of the human gene or expression of the human gene in yeast does not lead to a phenotype, then there is a requirement for a different type of assay. Indeed, fewer than 30% of human disease genes have a direct yeast orthologue (Yang et al., 2017). Here, using the specific example of the human *PTEN* gene, we described a general approach that can be used to identify yeast strains that can be used to measure the functionality of disease gene variants, a technique we name sentinel interaction mapping (SIM) (Fig. 1C). We demonstrate the use of both agar plate-based arrays of yeast and microtitre-plate liquid growth assays to classify variants. The key features of this technology are its generic nature, in that it should be applicable to a wide variety of human disease genes without requiring detailed knowledge of its biology, and its highly scalable nature, enabling rapid classification of hundreds of variants.

## RESULTS

### SIM – an overview

SIM can be used to measure the functionality of human disease gene variants in a procedure that identifies yeast strains that exhibit a growth phenotype upon overexpression of a human gene, and enables variants to be classified based on the extent to which they recapitulate this phenotype (Fig. 1D). The first step is to perform a synthetic dosage lethality (SDL) screen to identify candidate strains for further investigation. These potential strains are then validated in mini-array format, ideally using a small number of well-characterized LoF and wt-like variants in the gene of interest. Those strains that show a robust phenotype dependent on the presence of the functional disease gene can then be employed to rapidly test a large number of variants, using high-density mini-arrays or in high-throughput liquid growth assays. These experiments provide quantitative information on the level of function of each variant.

### SDL screening to identify yeast strains sensitive to overexpression of human genes

SDL screening (Kroll et al., 1996; Tong and Boone, 2006) is a variant of synthetic genetic array (SGA) analysis that identifies genetic interactions that result from the overexpression of a gene in each of the ∼4800 strains of the yeast deletion mutant array (DMA). In this proof-of-principle study, we aimed to identify yeast deletion strains that would exhibit a growth phenotype when the human *PTEN* gene product is overproduced. Loss of PTEN function has been implicated in a variety of human tumours (Li et al., 1997), non-cancerous neoplasia (Hobert and Eng, 2009) and neurological conditions such as autism (Napoli et al., 2012).

The *PTEN* gene encodes a phosphatidylinositol (PtdIns) (3,4,5)-trisphosphate [PtdIns(3,4,5)P3] phosphatase, which acts on the 3′ phosphate group of the inositol ring (Worby and Dixon, 2014). *PTEN* does not have a direct yeast homologue and its usual substrate PtdIns(3,4,5)P3 is not present in yeast. *PTEN* overexpression did not affect growth of wt yeast (Fig. S1A), suggesting that yeast physiology is sufficiently robust to buffer against PTEN action in yeast.

A query strain (Y7093) containing a plasmid that overexpressed *PTEN* from the yeast *GAL1* promoter was mated to the DMA. The resulting diploids were sporulated to generate haploid progeny and *PTEN* expression was induced by plating on galactose media. We simultaneously generated a control array from the same germination step by plating cells on media containing 5-fluoroorotic acid (5-FOA) to force counter-selection of the *PTEN*-expressing plasmid. Three replicates of each plate were analysed.

We quantified the growth of deletion strains overexpressing *PTEN* using the program Balony (Young and Loewen, 2013). We identified a strain as a potential ‘hit’ if it exhibited a statistically significant growth defect (*P*<0.05) relative to its corresponding control strain, and the extent of that growth defect was beyond an experimentally defined cut-off. The initial analysis indicated 324 potential hits, although from previous experiments we assumed that a significant proportion of these hits would be false positives due to statistical noise and the unpredictable growth of certain deletion mutants. It is also likely that a number of these potential hits would not be experimentally useful as the extent of the growth phenotype would be too small to repeatedly measure accurately. To identify genuine interactions, the entire screen was repeated to find hits that reproduced the growth phenotype (Fig. S1B). We additionally performed a control screen using an empty vector in place of the *PTEN*-expressing plasmid to help identify false positives. We also performed a screen with the catalytically inactive C124S variant of PTEN (Myers et al., 1998), which would not be expected to show the same genetic interactions as wt PTEN if those were indeed reporting on the enzymatic activity of PTEN (Table S1).

### Validation of sentinel strains

**Fig. 2.**
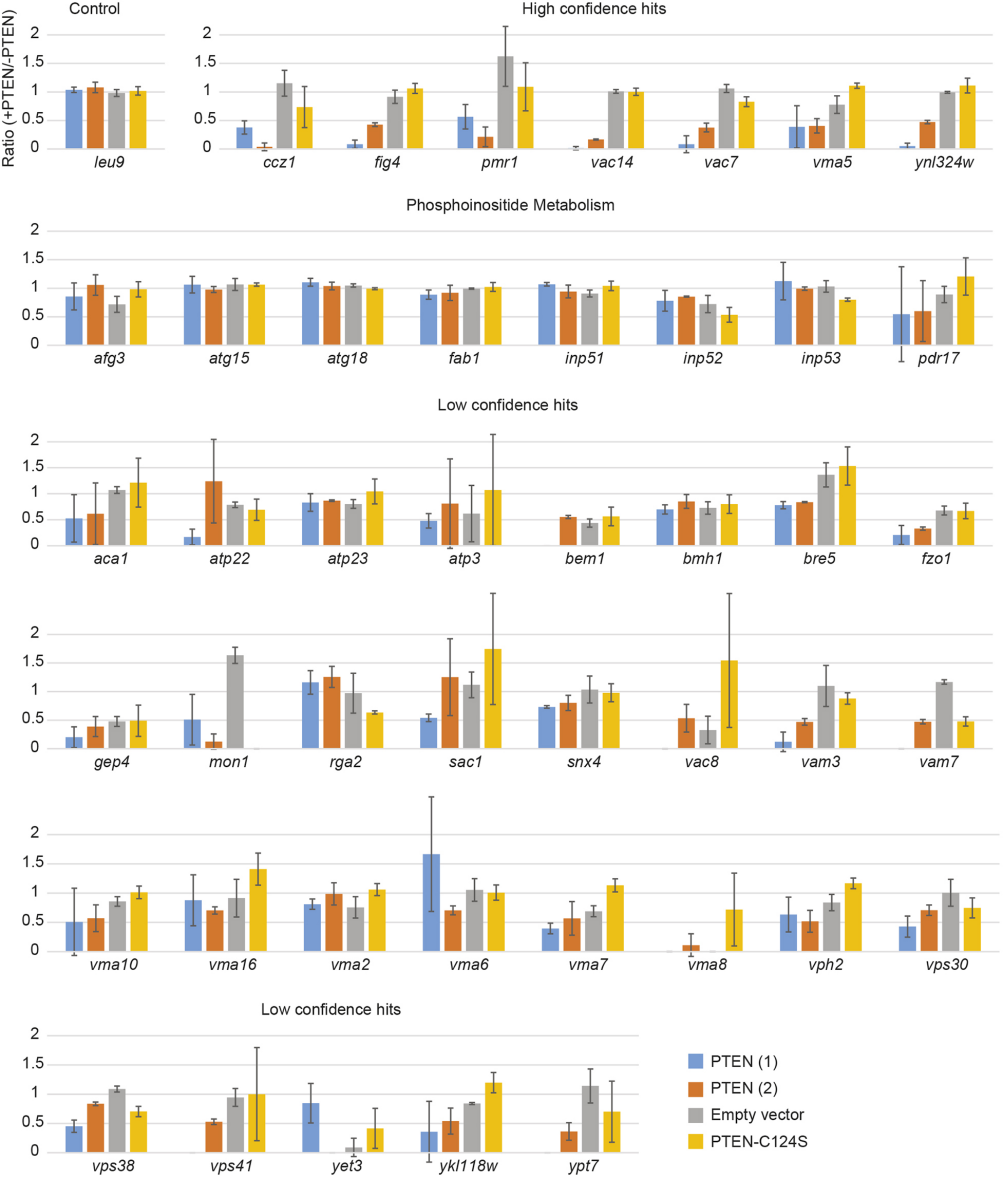
**Candidate yeast deletion strains for sensitivity to PTEN overexpression.** Mean ratio of normalized colony size (+PTEN/−PTEN) for the indicated deletion strains. Error bars show s.d. Each measurement is the average of three ratios.

Next, we constructed yeast mini-arrays using the candidate ‘sentinel’ strains identified from the SDL screens (Fig. 2). In these experiments, instead of testing the entire DMA, we selected a subset of strains to analyse with greater precision. This served two purposes. First, it would enable us to verify the results of the SDL screens with a higher degree of statistical rigour by increasing the number of replicates measured. Second, it would serve to test the feasibility of measuring the activity of multiple PTEN variants in a high-throughput manner.

Our SDL screens identified seven strains with a high degree of confidence (*ccz1*, *fig4*, *pmr1*, *vac14*, *vac7*, *vma5* and *ynl324w*); each of these strains showed a statistically significant growth defect upon overexpression of PTEN in all replicates in the original SDL screens. Interestingly, several of these strains are deletion mutants in genes that have roles in PtdIns3P metabolism. This suggests that PTEN activity in yeast involves phosphoinositides, similar to its role in higher organisms. We added a further 38 strains to our shortlist, adding low-confidence strains (four to five out of six replicates are hits between the two screens) and strains that were not hits but are involved in phosphoinositide metabolism, in case variants otherwise impacted these pathways. We also included the *leu9* strain as a control, which appeared to be unaffected by PTEN expression.

**Fig. 3.**
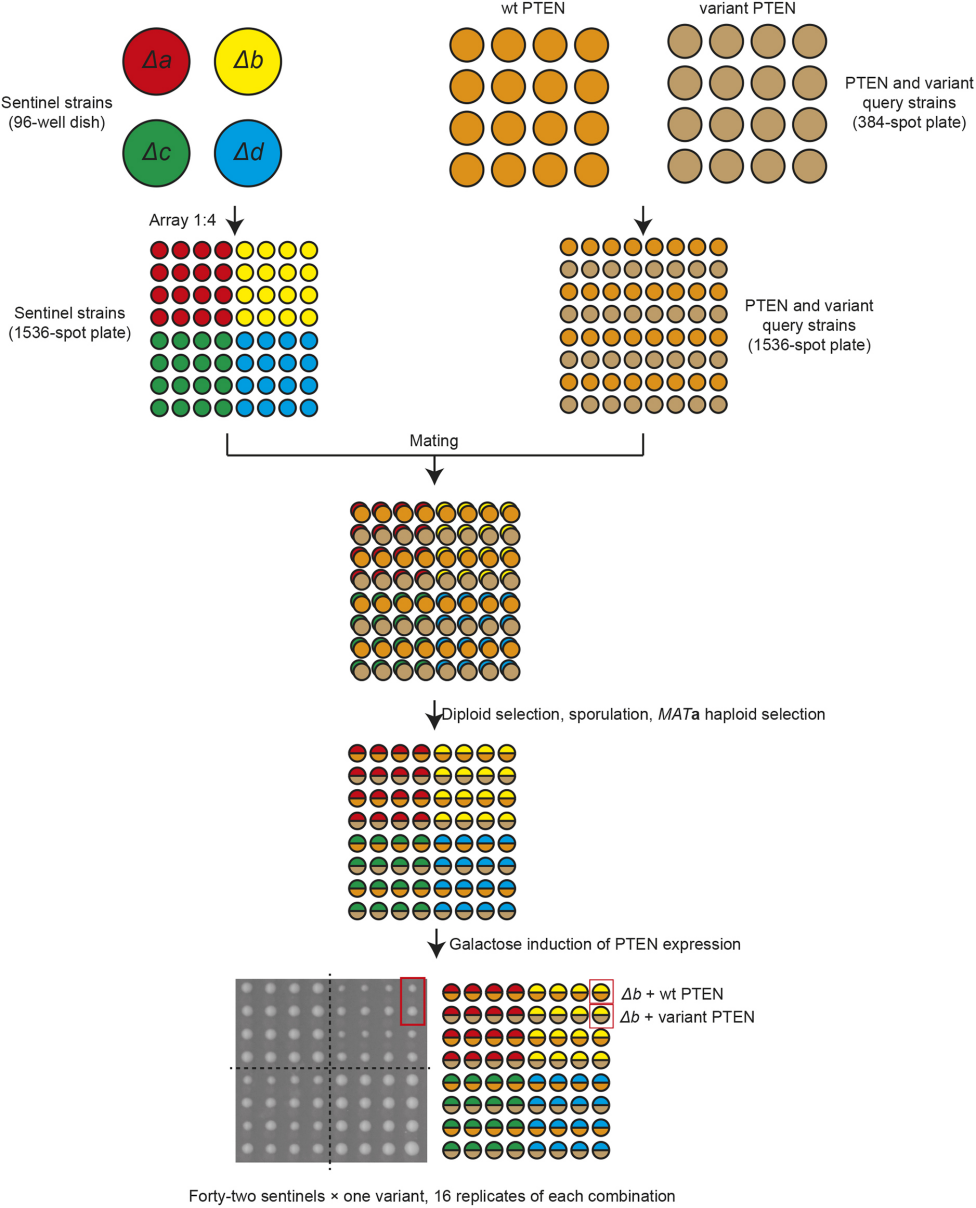
**A mini-array of 44 yeast strains expressing 42 PTEN variants.** Experimental overview. For simplicity, only four of the 44 deletion strains are shown. Plates are arrayed at a density of 1536 spots per plate.

The mini-arrays were constructed such that each deletion strain was measured in 16 replicates, with the exception of the control strain *leu9*, which was present 64 times (Fig. 3). These deletion arrays were then mated to a plate of query strains consisting of alternating rows of the strain Y7093 expressing either wt *PTEN* or one of five *PTEN* variants. To test the ability of sentinels to differentiate between function and non-functional PTEN we used the well-characterized LoF variants C124S and G129R (Furnari et al., 1998; Maehama and Dixon, 1998; Myers et al., 1997), in addition to an empty vector control. We also tested the variants T135I and T78A, two variants that are predicted to have low impact on PTEN function (Lek et al., 2016).

Images were collected of each mini-array plate and analysed using Balony. We made modifications to the program to accommodate the mini-array format, specifically adding the option to define control spots within the same plate, rather than corresponding spots on a separate plate. We also added functions to summarize the results from multiple screens and generate data tables collating these results.

**Fig. 4.**
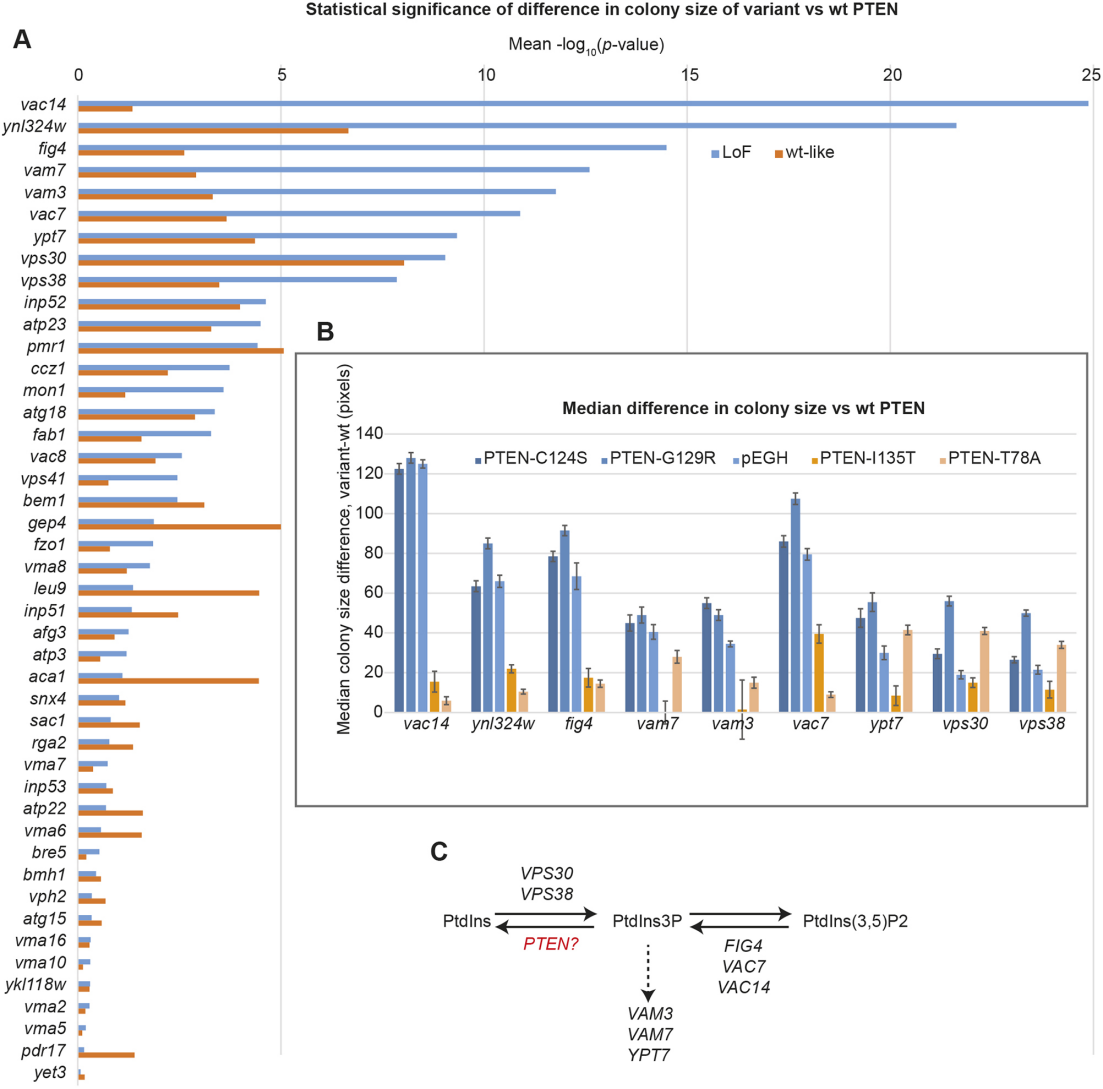
**Verification of sentinel sensitivity by mini-array analysis.** (A) Colony sizes of the indicated strain were compared in the presence of wt PTEN to LoF variants (blue) or wt-like variants (orange). The *P*-value is based on a two-tailed unpaired Student's *t*-test of colony pixel areas. (B) Median difference in colony size. Each sentinel/variant colony is paired with a wt PTEN control. Error bars show s.d.

We ranked the deletion strains based on the statistical significance of difference in colony sizes when expressing wt PTEN compared with the LoF PTEN variants and vector control. The nine strains with the highest statistical significance all yielded an average *P*-value of <10^−7^ across these three conditions (Fig. 4A). The significance of colony size difference was considerably reduced when wt PTEN was compared with the predicted low-impact T78A and I135T variants. This was also reflected in the median difference in colony size, with the LoF variants showing larger differences in colony size than the low-impact variants (Fig. 4B). There were considerable differences in the characteristics of the sentinels, with a greater difference in colony size predictably corresponding to higher statistical significance. Additionally, the strains that do not have a growth phenotype in the absence of PTEN expression (*vac14*, *fig4* and *ynl324w* – see Table S1 ‘Control’ column), have the highest statistical significance. This suggests that ‘healthy’ strains may make the best sentinels, presumably because of their predictable growth rate, which would be unaffected by suppressor mutations.

The identity of the genes deleted in these strains provided a clear insight into the biological activity of PTEN in yeast. In mammalian cells, PTEN acts as a lipid phosphatase with its preferred substrate PtdIns(3,4,5)P3, which it converts to PtdIns(4,5)P2. However, although PtdIns(3,4,5)P3 is not found in yeast, a secondary substrate of PTEN, PtdIns3P, is (Maehama and Dixon, 1998). It therefore seems likely that PTEN in yeast catalyzes the conversion of PtdIns3P to PtdIns (Fig. 4C). Five of the strains are knockouts of genes directly involved in synthesis of PtdIns3P (Jin et al., 2008; Kihara et al., 2001; Rudge et al., 2004), either from PtdIns [*VPS30* and *VPS38*, encoding members of the type II phosphoinositide 3-kinase (PI3K) complex] or from PtdIns(3,5)P2 (*FIG4*, *VAC7* and *VAC14*, encoding members of the PAS complex). Meanwhile, *VAM3*, *VAM7* and *YPT7* encode proteins involved in vacuole fusion downstream of PtdIns3P (Haas et al., 1995; Sato et al., 1998). Therefore, these strains are likely to be sensitized to changes in PtdIns3P levels, such that induction of PTEN activity would lead to a growth defect. However, wt cells are not sensitized in such a way, thus explaining why overexpression of PTEN in wt yeast has no effect on growth (Fig. S1A).

In this case, knowing the function of PTEN in mammalian cells made it simple to formulate a hypothesis for its activity in yeast cells. However, had PTEN been an uncharacterized gene, the genetic interactions in yeast would have provided clues to its function. This demonstrates how overexpressing a heterologous protein in the yeast deletion collection and mapping genetic interactions could provide insights into the roles of uncharacterized human genes.

### Construction of yeast mini-arrays to measure activity of PTEN variants

To demonstrate the ability of these sentinels to report on PTEN activity, we sought to measure the strength of the genetic interaction of each deletion mutant with 100 PTEN variants, comprising a mixture of disease-associated variants and population controls. This was done as part of a larger study using multiple model organisms to functionalize PTEN variants found in autism spectrum disorder, cancer and PTEN hamartoma tumour syndrome (Post et al., 2020). Based on the results of the mini-array experiments described above, we selected the eight strains that demonstrated repeated sensitivity to functional PTEN activity (*vac14*, *fig4*, *vac7*, *vam3*, *vam7*, *ypt7*, *vps30* and *vps38*). The dubious open-reading frame *YNL324W* overlaps the *FIG4* gene, so the *ynl324w* strain was excluded as its phenotype is presumably caused by disruption of *FIG4*.

**Fig. 5.**
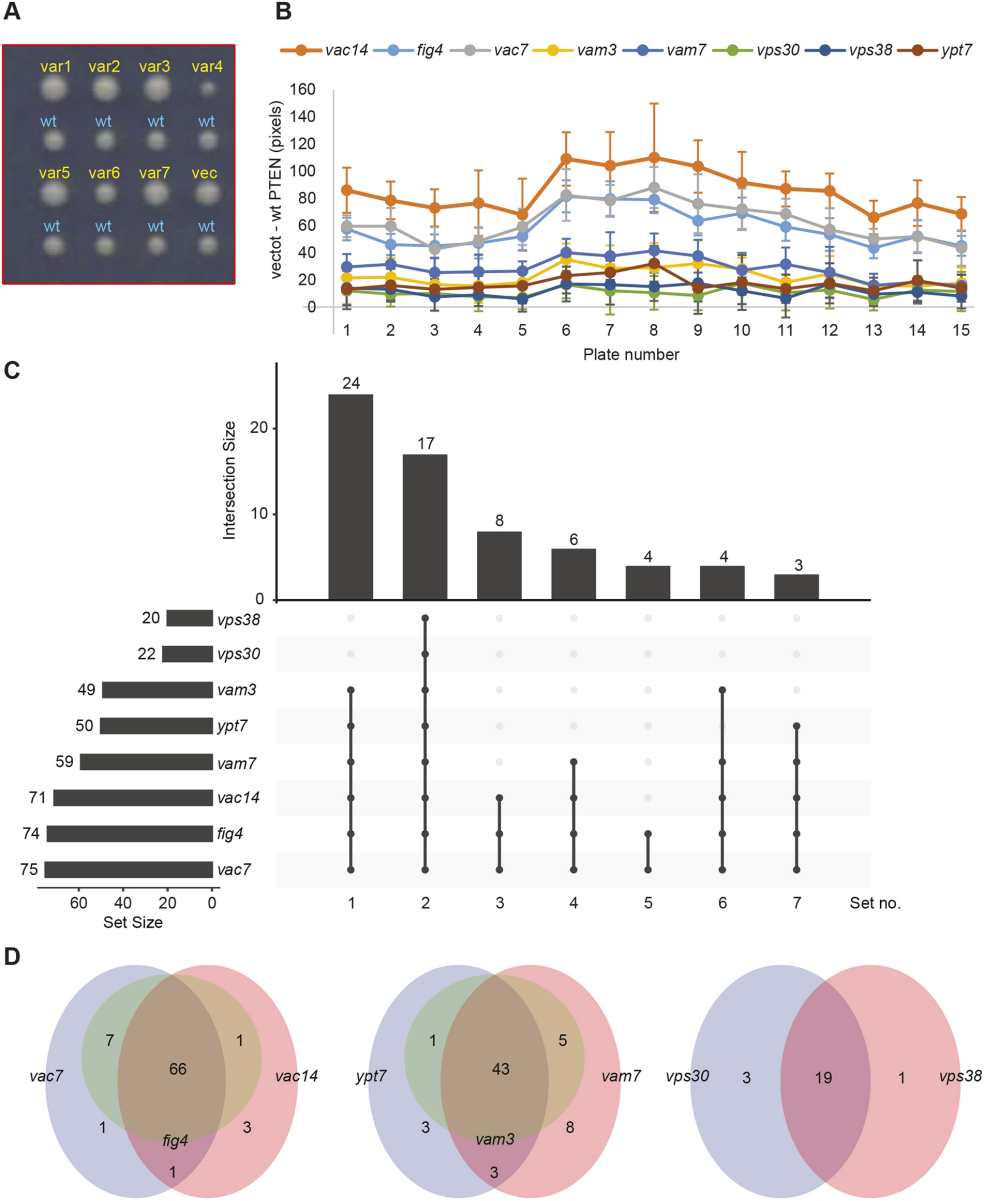
**High-throughput functionalization of 100 PTEN variants.** (A) Sample section of mini-array plate image showing seven different PTEN variants expressed in *fig4* yeast. Each variant spot (yellow) is coupled with a wt PTEN control spot (cyan). (B) Dynamic range of sentinel strains. The median difference in area between vector control and wt *PTEN*-expressing strains is shown for each of the 15 mini-array plates. Error bars show s.d. (C) Intersection between sentinels based on number of variants reported as significantly different from wt. Contents of sets are shown in Fig. S3. (D) Overlap of significantly different variants within sentinel groups: (left to right) PAS complex, PtdIns3P downstream targets, PI3K type II complex.

In order to improve the high-throughput nature of the assay, we increased the number of variants analysed per plate from one to seven. To simulate the effect of a complete LoF variant, we included an empty vector control on each plate, thus making it possible to define the dynamic range of each sentinel. The process for the construction of the high-throughput mini-arrays is shown in Fig. S2. Once again, each spot analysing a PTEN variant was paired with an adjacent spot expressing wt PTEN (Fig. 5A), which we determined to be critically important for reducing spot-size measurements between plates (Fig. 5B). These variations likely result from inconsistencies in the robotic pinning steps and variations that result from the image capture stages.

The PTEN variants were arrayed across 15 plates at a density of 1536 spots per plate. This enabled each variant to be analysed in eight sentinel strains, with 12 replicates of each variant/sentinel combination. We scored each spot by measuring its area in pixels, then subtracting the area of the adjacent wt PTEN control spot.

To determine the relative performance of the sentinel strains to detect PTEN functionality, we determined which variants each sentinel identified as being significantly different from wt PTEN with a *P*-value <0.05. We then analysed the resultant groupings of sentinels using the R package UpSetR (Conway et al., 2017). This separated the sentinels into roughly three groups (Fig. 4C). The most sensitive sentinels were *vac14*, *fig4* and *vac7*, which each predicted 71-75 variants where the median spot size was different to the control spot size with a *P*-value <0.05. Next were *vam3*, *vam7* and *ypt7*, which predicted 49-59 variants. Finally, *vps30* and *vps38* were relatively insensitive, each predicting 20 or 22 variants. Interestingly, these groupings correspond to the different cellular pathways in which these genes are found (Fig. 4C). We defined seven sets of variants based on the sentinels in which they were shown to be different to wt (Fig. S3A). Generally, the sets that contained the most sentinels were those where the growth difference was greatest, reflecting a larger loss of function. Similarly, the sets that contained fewest sentinels represented a smaller loss of function, which only the most sensitive sentinels could detect. As might be expected, the sensitivity of sentinels reflected the dynamic range of the growth defect between cells expressing wt *PTEN* and empty vector, with *vac14*, *fig4* and *vac7* showing the largest difference in spot size.

Within each grouping, there was a high degree of consistency between the variants identified as different (Fig. 5D). The vast majority of variants that were classified as non-wt by the less-sensitive sentinels were also categorized this way for the more-sensitive sentinels (first and second columns, Fig. 5C), consistent with the notion that these strains are reporting on the same activity at different levels of sensitivity.

Interestingly, we noted that our approach also enabled the identification of gain-of-function variants, something that is often not possible with complementation assays (Sun et al., 2016). The 4A variant was specifically constructed to remove inhibitory phosphorylation of PTEN at four positions by conversion of serine or threonine residues to alanine (Vazquez et al., 2000). This results in a constitutively active protein with phosphatase activity greater than that found in wt PTEN. The specificity of this effect is shown by the C124S-4A variant, which has an additional mutation in the active site of the phosphatase domain and results in no detectable enzyme activity. Two of our most sentinel strains (*fig4* and *vac7*) were able to detect the gain-of-function activity of the 4A variant (Fig. S3B), i.e. these strains had a more-severe growth defect when expressing 4A compared with wt *PTEN*. The *vac14* strain did not detect increased PTEN activity above wt levels, despite having a greater dynamic range for LoF mutations (Fig. 4B). It is likely that the growth defect in *vac14* caused by expression of wt *PTEN* is so severe that any additional activity cannot be reliably detected in this assay. This suggests that these sentinels cover a different functional range of PTEN activities and highlights the utility of using multiple sentinels to assay variant function.

### Analysis of variant function by liquid growth assay

Next, we investigated the extent to which our assays could provide quantitative measures of PTEN activity, rather than a simple ‘functional/non-functional’ output. To do this, we used high-throughput liquid growth assays, which would be expected to provide more-sensitive measures of growth rate due to the measurement of growth at multiple points during a time course.

**Fig. 6.**
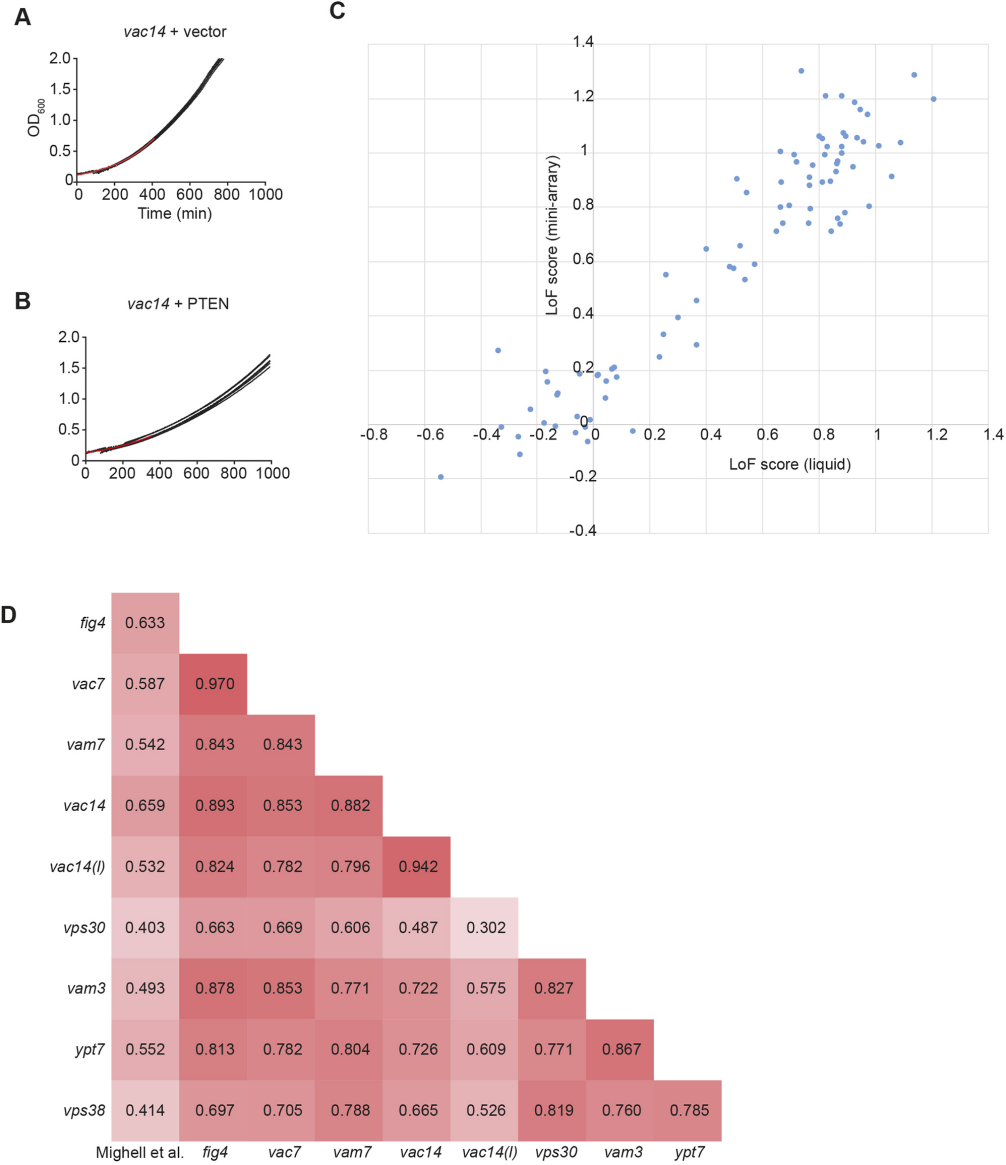
**Validation of variant functionalization using liquid growth assays.** (A,B) Curve fitting of logarithmic-phase growth of *vac14* yeast with or without wt *PTEN* expression. The red curve is the exponential fit to the first 6 h of the average of 12 sets of A_600_ measurements (black). (C) Scatter plot showing LoF scores for 87 PTEN variants on solid media (*y*-axis) versus liquid growth assays (*x*-axis). (D) Pearson correlation matrix of different PTEN variant assay methods. LoF scores for 87 variants were aligned and correlation coefficients calculated with GraphPad Prism.

Using an automated plate reader, the growth rate of yeast strains can be measured via the increase in absorbance at 600 nm (A_600_) over time. Following calibration and fitting to an exponential function, a rate constant *k* can be determined. To measure the growth rate of each of the variant-expressing strains, 96-well dishes were seeded with one variant per column, giving eight technical replicates. This allowed for the measurement of ten variants per plate, with two columns reserved for wt PTEN and an empty vector control. As the *Δvac14* sentinel seemed to be the most sensitive to PTEN expression (Fig. 4B), we used this strain in these assays (Fig. 6A,B). We determined the growth rate of a total of 87 variants. To directly compare the results between the liquid growth and mini-array assays, we calculated an LoF for each variant/sentinel combination.

For mini-array-based assays, we used the formula:
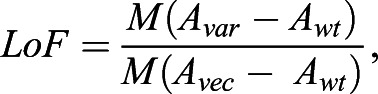
where the upper term is the median difference in spot size between the variant spot and its corresponding wt control, and the lower term is the median difference in spot size between the vector control and its corresponding wt.

Similarly, for the liquid growth assays the formula used was:
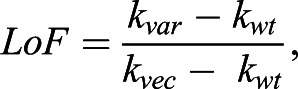
where *k_var_*, *k_wt_* and *k_vec_* are the rate constants for the variant, wt control and vector control from a given microtitre-plate. This results in the scaling of scores, such that wt-like variants have a score of ∼0 and complete LoF variants a score of ∼1.

The growth rates in liquid data correlated closely with the colony size on agar plates (Fig. 6C), with an r^2^ value of 0.887, suggesting that the mini-array data can indeed be used to provide quantitative information on the level of PTEN activity of a variant. We found close agreement in the intermediate range of LoF scores (i.e. ∼0.2-0.8) for both approaches, suggesting that these assays are sensitive enough to identify partial LoF variants.

Interestingly, we were able to demonstrate the gain-of-function activity for the 4A mutant in *vac14* cells when using the liquid growth assay (LoF=−0.265; *P*<0.0001), which we did not detect in the mini-array analysis (LoF=−0.0735; *P*=0.72). This is likely because the growth rate determined in liquid is more precise and hence can reliably detect smaller relative growth changes.

### Comparison with existing variant analyses

As a further step to validate the reliability of our assay, we compared our analysis with another yeast study that characterized PTEN variants (Mighell et al., 2018). Here, a yeast strain overexpressing the human PI3K p110α was used, which causes a growth defect in wt yeast (Cid et al., 2008; Tugendreich et al., 2001). This phenotype can be reversed by overexpression of wt human *PTEN* (Rodríguez-Escudero et al., 2005). In this analysis, ∼8000 PTEN variants were screened for rescue of the p110α growth phenotype in a massively parallel assay. Variants were given a score that corresponded to whether they were wt-like or likely damaging. To compare these results with our own variant analysis, we converted their fitness metric to a similar scale to our LoF score. These values were then used to calculate a Pearson correlation matrix, which also comprised our liquid growth assay data and the eight mini-array sentinels (Fig. 6D). A total of 82 variants were compared.

The highest correlation between any two assays was found for the *vac7* and *fig4* mini-arrays (r=0.970), closely followed by the *vac14* mini-array and the *vac14* liquid growth assay (r=0.942). When comparing the p110α-PTEN data to our assays, the highest correlations were found with the *vac14* and *fig4* mini-arrays, with Pearson correlation coefficients of 0.659 and 0.633, respectively. This broad agreement between two conceptually very different assay systems demonstrates the suitability of the SIM approach for variant functionalization. The high degree of correlation between sentinels also supports the robustness of the SIM approach for quantitatively measuring the effects of variants on gene function.

We suspected that our assays may have an advantage in their ability to provide reliable quantitative information while also offering high-throughput capabilities. When we inspected the data from the p110α overexpression study, we noted that ∼10% of variants were not scored with high confidence. For example, the G129R mutation is well characterized as having a complete loss of PTEN function (Furnari et al., 1997; Wozniak et al., 2017). Yet in the p110α study it was not classified as likely damaging with high confidence. However, all eight of our sentinel strains identified G129R as different to wt (*P*<0.05), as did our *vac14* liquid growth assay.

### SIM as a predictor of variant pathogenicity

While the SIM assays demonstrated a clear ability to predict PTEN function in yeast, we were interested to see the extent to which this corresponded to the pathogenicity of annotated variants. To evaluate the predictive value of the sentinels, we built machine-learning models based on logistic regression. For model training, we curated reference data for each variant using clinical annotations from ClinVar as well as information from the COSMIC and gnomAD databases (Table S2). A separate model was built for each sentinel to predict the pathogenicity of PTEN variants. For comparison, we also built models using data from Mighell et al. (2018) and from PolyPhen2, a widely used computational prediction algorithm (Adzhubei et al., 2010). The performance of each of these models is listed below (Table 1).

**Table 1.**
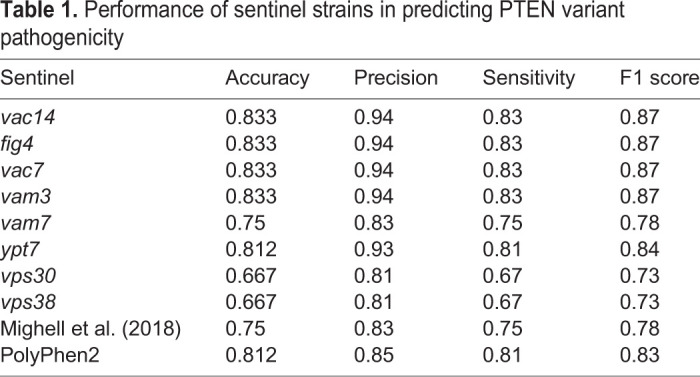
Performance of sentinel strains in predicting PTEN variant pathogenicity

There was a clear correlation between the sentinels that were most sensitive in predicting PTEN function and pathogenicity. For example, the *vac14* sentinel, which was able to distinguish 75/100 variants from wt PTEN, had an F1 score of 0.87, whereas *vps30*, which only distinguished 22/100 variants, had a score of 0.73. We also compared the sentinels to the p110α overexpression assay (Mighell et al., 2018) and found that SIM performed as well as, if not better than, this assay. When compared to PolyPhen2, the best-performing sentinels demonstrated similar accuracy (0.833 versus 0.812), but higher precision (0.94 versus 0.85) than this bioinformatics method, highlighting the advantages of an *in vivo* approach.

As the model performance is dependent on the reference data containing accurate information on the pathogenicity of a given variant, it is possible that these scores may underestimate the predictive power of sentinels; for example, if a variant is annotated as being benign when it is in fact deleterious.

## DISCUSSION

In this study, we have shown that the repertoire of human gene variants that can be analysed in yeast can be extended beyond those genes for which direct orthologues exist. Using SDL screening, thousands of candidate strains can be rapidly assessed to find deletion mutants that are sensitive to overexpression of the human gene. Upon validation of the screen results, these sentinel strains can then be employed to test a panel of variants. Although in this study we found that different sentinel strains largely coincided on identifying LoF variants, it is likely that for other human genes, different sentinel strains could report on different aspects of a protein's function. In this scenario, different deletion strains would be sensitized to the function of specific domains within a human protein. Hence, using multiple sentinels to characterize variants either by mini-array or liquid growth assays would facilitate functionalizing all domains of a protein.

Furthermore, SIM has the potential to discriminate between LoF and gain-of-function mutations, which could be crucial in identifying variants for which pathogenicity is a result of overactivity, as is found with many oncogenes. This is possible because SIM is based on reduced growth of yeast the closer a variant is to wt function, providing scope to detect protein function above wt levels. With complementation testing, this is not possible if expression of the human gene completely restores the growth phenotype of the yeast deletion strain. In this case, gain-of-function mutations may still lead to a deleterious phenotype, as has been observed with variants of the superoxide dismutase gene *SOD1* (Bastow et al., 2016). However, care must be taken in interpreting apparent gain-of-function variants in yeast. For example, hyperactive complementation can be observed with certain variants of human genes in cases where the wt gene does not fully rescue the growth phenotype of the analogous yeast deletion strain (Weile et al., 2017). However, these variants tend to be deleterious in humans and the apparent increased activity is likely a spurious consequence of expression in yeast. The same caveat likely also applies to SIM.

An additional advantage of using SDL screening to identify sentinel strains is that it is an unbiased approach requiring no prior knowledge of the function of the gene being investigated. Indeed, the nature of sentinel strains can actually provide insights into human gene function; the sentinels identified in this study pointed to the well-described role for PTEN in phosphoinositide biology. Given the highly annotated nature of the yeast genome, SIM has the potential to reveal hitherto unknown roles for human genes, facilitating more rapid characterization of diseases. There is indeed precedence for the role of yeast in this area. Studies in yeast have shed light on the mechanism of action of alpha-synuclein in neurodegenerative disorders such as Parksinon's disease. (Cooper et al., 2006; Elden et al., 2010; Gitler et al., 2009). SIM could be especially useful in the case of rare diseases where there is often uncertainty about the causative disease gene and its normal function. The relatively low cost and simplicity of the SIM approach may provide useful insights into rare genetic disorders for which funding is an otherwise limiting factor.

We have shown that variants can be analysed using either agar plate-based arrays or high-throughput liquid growth assays. Both methods are instructive, and the choice of which to use will depend upon the resources of the investigating laboratory and the specific goals of the project. The techniques have different advantages. The liquid assays likely provide higher precision due to the directness with which growth is measured. However, most laboratory plate readers will only analyse a single plate at a time, thus imposing something of a bottleneck if a large number of variants are to be analysed. Using a colony-arraying robot is advantageous in that a large number of plates can be pinned in a single session, thus allowing for greater numbers of variants to be screened in a single experiment. However, as the measurement of growth is somewhat indirect, greater statistical noise is found in the measurements. This can be somewhat compensated for by measuring a sufficiently large number of replicates; the exact number to provide suitable accuracy may need to be determined experimentally depending on various factors including the dynamic range of the genetic interaction and the consistency of pinning by the particular robot being used.

Beyond the approach described here, there are modifications to the SDL protocol that could be employed to broaden the scope of SIM assays. For example, here we have only screened the haploid deletion collection to identify sentinel strains. Other arrays, such as the temperature-sensitive collection of essential yeast genes could be screened. SIM could also be performed with *Schizosaccharomyces* *pombe*, which is as distant in evolution from humans as it is from budding yeast and captures human-related functions not well represented in budding yeast (Frost et al., 2012). While we used the popular *GAL* expression system in this study, other inducible systems could be used to drive expression of the human gene. Employing a titratable promoter such as the beta-oestradiol-inducible activator system (Gao and Pinkham, 2000) would open the door to testing a human gene over a wide range of expression levels to further extend the flexibility of the system. This would be especially useful if a human gene restricts growth of wt yeast but identifying sentinels is still required; for example, if the function of the human gene is unknown or if it has multiple functions and it is desirable to determine which variants affect which functions. It is also likely that for some human disease genes it will not be possible to identify sentinels; for example, if the human protein is not folded correctly in yeast or lacks critical unknown co-factors or binding partners. In such cases, it may be possible to express individual domains of the protein that could improve folding or solubility, and eliminate requirements for regulatory factors. Nevertheless, the additional time invested in devising an appropriate expression system would still likely be repaid by the speed and cost-effectiveness of high-throughput variant characterization in yeast.

## MATERIALS AND METHODS

### Yeast strains and PTEN variants

Strain Y7093 is a variant of Y7092 (Tong and Boone, 2006) with the *NatR* cassette integrated into the *TRP1* locus to enable selection of diploids on YPD (+G418/clonNAT) medium during the SGA process. The human *PTEN* gene was amplified by PCR, then co-transformed into Y7093 along with pEGH (Zhu et al., 2001) cut with SacI. Gap repair resulted in the production of plasmids expressing PTEN from the *GAL1-10* promoter. Site-directed mutagenesis by PCR was used to create PTEN variants, which were recombined into pEGH as above.

### SDL screens

All screening steps were performed using a singer RoToR HDA colony-arraying robot. All steps were performed at a density of 1536 spots per plate unless otherwise indicated. PTEN was overexpressed in the yeast deletion collection essentially as described previously (Young and Loewen, 2013; Fig. S4). Briefly, Y7093 expressing PTEN under the *GAL1-10* promoter was mated with the yeast DMA at a density of 1536 spots per plate. Following selection on YPD+G418+clonNAT medium, diploids were sporulated for 10 days at 25°C. Haploid *MATa* cells were germinated on SD-His/Arg/Lys/Ura (HURK) medium containing G418, canavanine and thialysine with 2% dextrose. To generate deletion mutants overexpressing PTEN, two rounds of plating on HURK+G418/canavanine/thialysine with 2% raffinose, 2% galactose were performed. To generate a control data set, haploids were first plated on SD-His/Arg/Lys (HRK)+G418/canavanine/thialysine with 2% raffinose, 2% galactose with 1 mg/ml 5-fluoro-orotic acid to counter-select the PTEN plasmid. This was followed by one round of plating on HRK+G418/canavanine/thialysine with 2% raffinose, 2% galactose. After 24 h of growth, plates were scanned and analysed using Balony software.

### Yeast mini-arrays

The overall protocol for screening variants by mini-array analysis was the same as for the SDL screens with some adjustments. Two mini-arrays were used in this study: one to validate candidate sentinel strains (‘mi-1’) and one to screen 100 PTEN variants (‘mi-2’). In the place of the yeast deletion collection, a set of either 45 (mi-1, Fig. 3) or eight (mi-2, Fig. S2) deletion strains were used. These were arrayed in a 96-well dish with either two (mi-1; apart from *leu9*, eight copies) or 12 (mi-2) copies of each deletion strain. This was then arrayed to an agar plate at 1536 spots per plate by copying each well of the 96-well dish to a 4×4 array of spots. In the place of the single query strain, a query array was constructed. For mi-1, this consisted of alternating rows of Y7093 expressing either wt or variant PTEN. Two 384-spot plates were combined to make a single 1536-spot plate. For mi-2, sixteen 96-spot plates were combined. Eight of these plates were arrays of Y7093 expressing wt PTEN. The other eight were arrays of Y7093 expressing a PTEN variant or a vector control.

### Yeast liquid growth assays

Liquid growth assays were performed with a BioTek Epoch plate reader, which was calibrated by taking multiple readings of serially diluted yeast cultures and fitting the absorbance measurements to a polynomial equation of the form *y*=a(A_600_)^3^+b(A_600_)^2^+c(A_600_)+d (Jung et al., 2015), where *y* is the relative number of cells. Through repeated measurements, we determined that the maximal growth phase of logarithmic growth occurred for the first 6 h of growth after initial dilution. When fitted to an exponential function of the form y=a*e^kx^*, the rate constant *k* is a measure of the rate of growth of that strain.

Strains were copied from –Ura/2% dextrose-rich media plates to –Ura/2% raffinose, 2% galactose (-Ura/Raf/Gal) plates for 2 days at 30°C. These plates were used to inoculate 2 ml cultures of -Ura/Raf/Gal liquid medium, which were grown overnight at 30°C. The following morning, cultures were adjusted to an A_600_ of 0.25 and grown for 6 h to ensure that cells were in log phase. At this point, cultures were adjusted to an A_600_ of 0.125 and used to seed wells of a 96-well plate. Each well contained 200 µl of culture. The insides of lids were washed for 15 s in 0.05% Triton X-100, 20% ethanol and air dried to reduce condensation (Jung et al., 2015). Plates were then sealed with Parafilm M to eliminate evaporation. Plates were incubated with shaking at 300 rpm at 30°C. A_600_ recordings were taken every 4 min. Relevant data points were fitted to an exponential curve using GraphPad Prism using the mean normalized reading of eight technical replicates.

### Bioinformatics analysis

Images of SGA plates were quantified using Balony (Young and Loewen, 2013), as previously described. Heatmaps were produced using the Heatmapper web site (Babicki et al., 2016). Set intersections were determined and visualized using UpsetR (Conway et al., 2017) in RStudio (RStudio Team, 2015).

Logistic regression analysis was performed using the scikit-learn package (Pedregosa et al., 2011). A reference dataset was labelled using ClinVar data, with uncertain and conflicting interpretations unlabelled (Table S2). The reference dataset was divided into training and testing data in a 70-30 split. Accuracy, precision, recall and F1 scores were computed using the Metrics module from scikit-learn. Fivefold cross-validation was also performed and the average accuracy score reported. Scripts used are available on GitHub at https://github.com/jessecanada/Young_SIM_2019. Parameters were calculated as follows: Accuracy=(TP+TN)/(TP+TN+FP+FN); Precision=TP/(TP+FP); Sensitivity=TP/(TP+FN); F1-Score=(2TP)/(2TP+FP+FN) (TP, true positive; FP, false positive; TN, true negative; FN, false negative).

## Supplementary Material

Supplementary information
