# Supplementary material for: Sentinel interaction mapping – a generic approach for the functional analysis of human disease gene variants using yeast
Source: Dis Model Mech. 2020 Jul 8;13(7):dmm044560. doi: 10.1242/dmm.044560 (PMC7358137; doi:10.1242/dmm.044560)
Supplement: Supplementary information [file dmm-13-044560-s1.pdf]

Supplementary Information

Figure S1

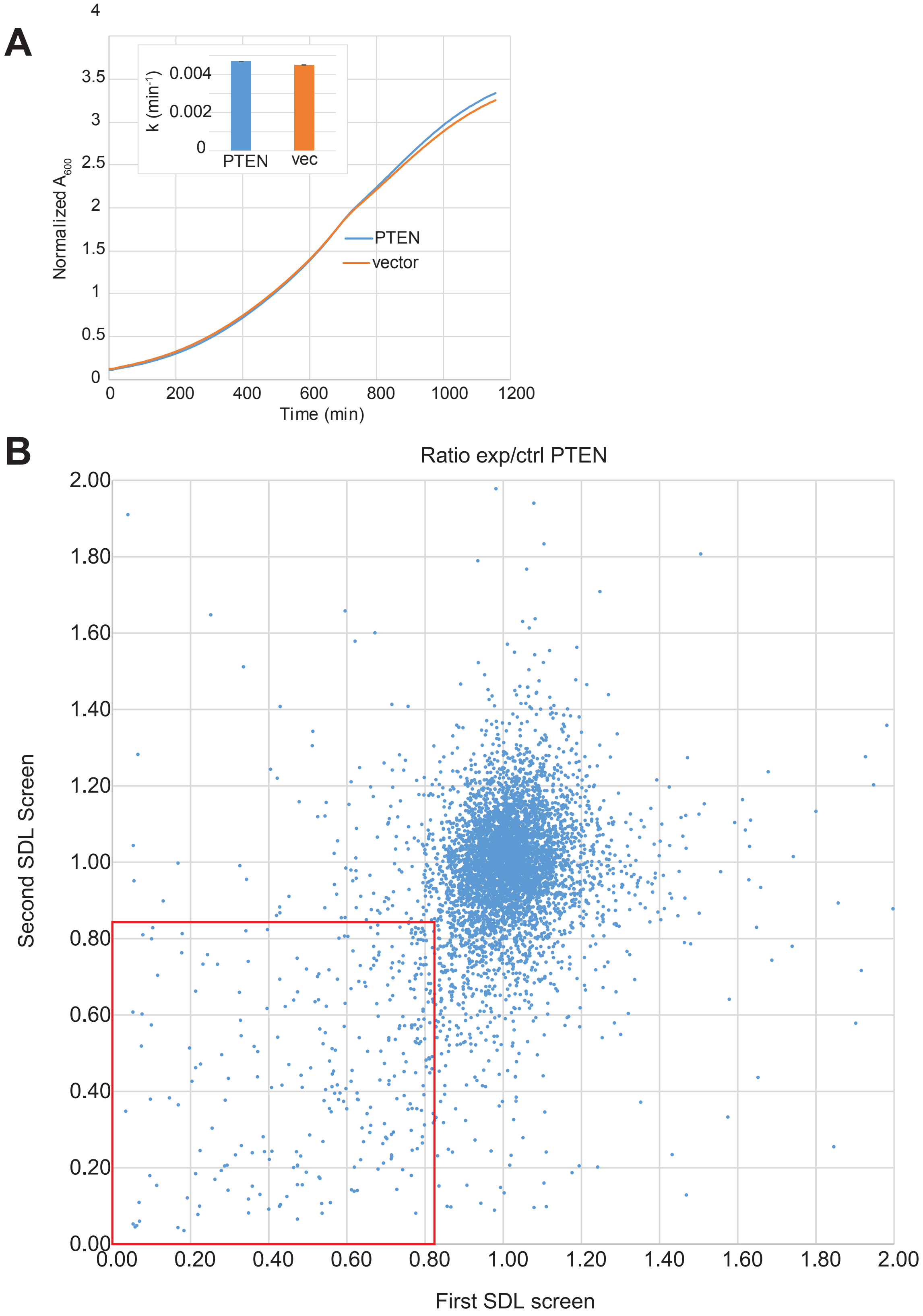

Figure S1. A. Overexpression of PTEN in WT yeast has no effect on growth. Growth of wt yeast with vector control or expressing WT PTEN was measured by liquid growth assays in SD + 2% galactose media at 30 °C. **B. Comparison of results from two SDL screens with wt PTEN.** The mean ratio (experimental spot/control spot) for each array position is shown for the first SDL screen (x axis) compared to the second SDL screen (y axis). The boxed area indicates genetic interactions (mean ratio <0.85) common to both screens.

Disease Models & Mechanisms • Supplementary information

Figure S2

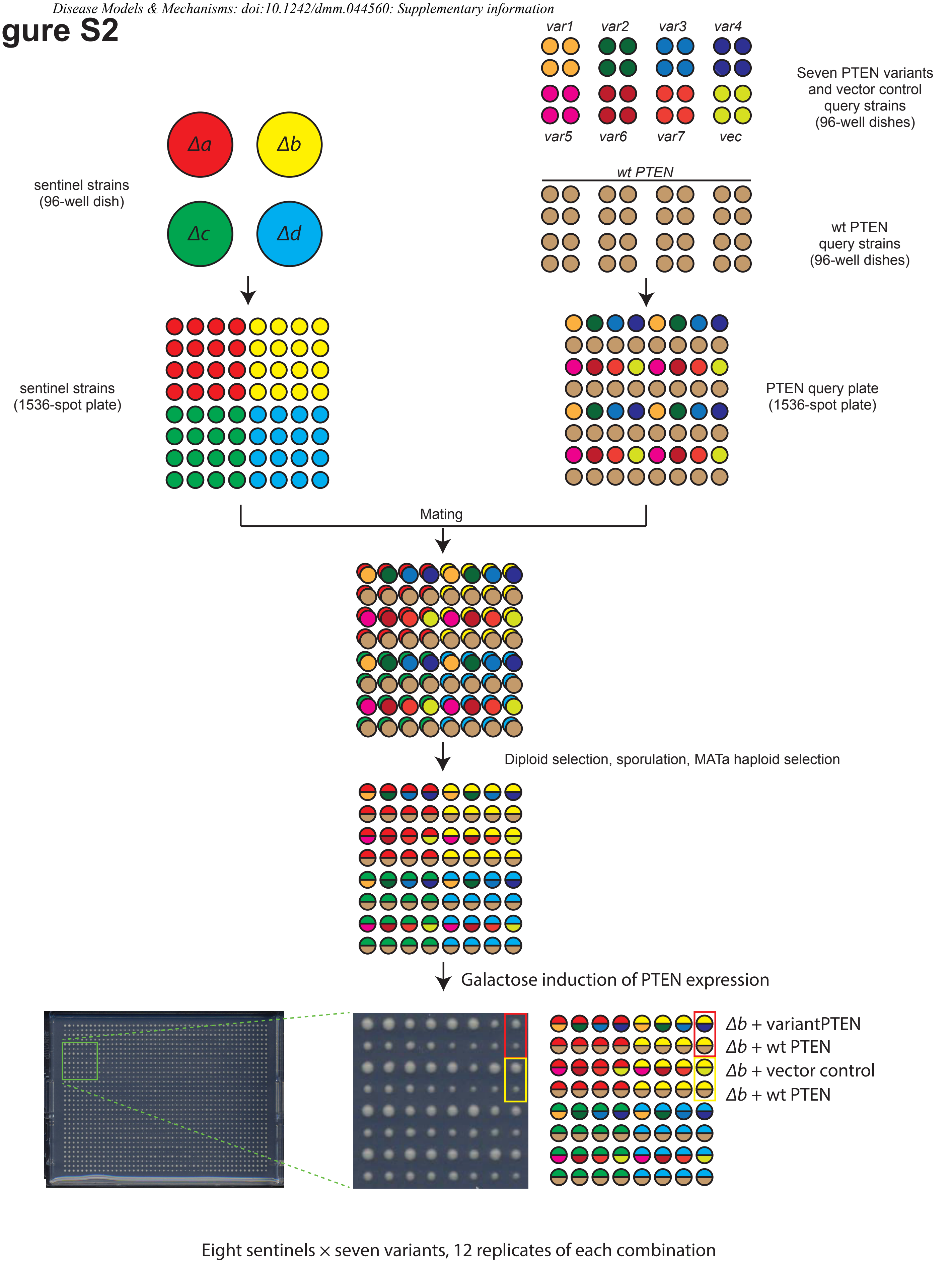

Figure S2. Schematic for SIM analysis on agar plates. A segment of a plate is shown for simplicity. Each plate analyzes seven variants and one vector control in eight different sentinel strains. Every sentinel/variant combination is present in twelve replicates.

Disease Models & Mechanisms • Supplementary information

Figure S3

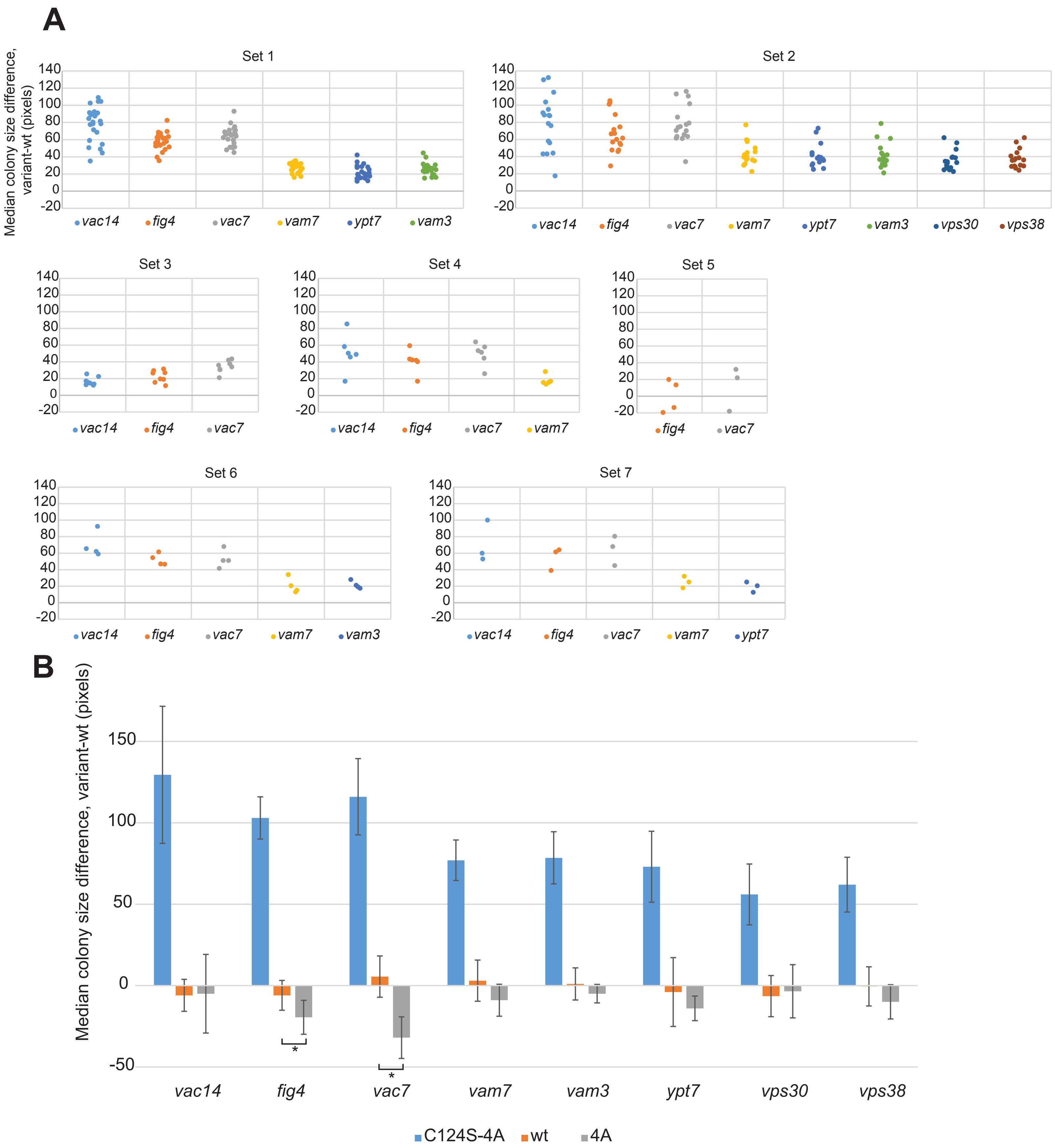

Figure S3. A. Composition of sentinel sets. Each set contains a number of variants that showed a significant growth defect compared to wt PTEN. Each spot represents a specific sentinel/variant pair. **B. Detection of gain-of-function variants.** Asterisks indicate $p<0.05$. Error bars indicate standard deviation of the difference between paired spots.

Figure S4

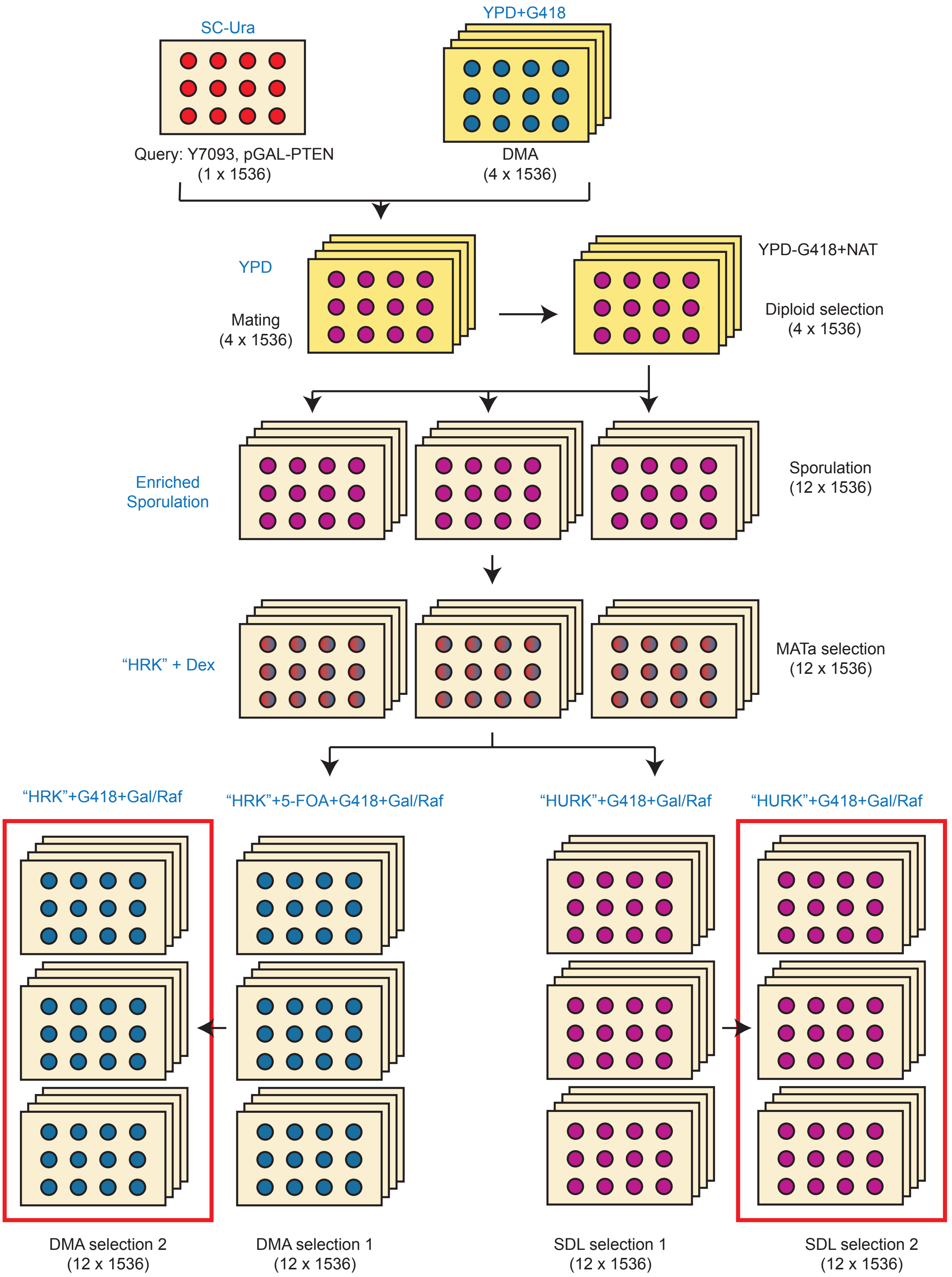

Figure S4. Schematic for SDL screening. Adapted from Young and Loewen, 2013. Growth media are indicated in blue. Numbers and array densities of plates are indicated in parentheses. Plates boxed in red are imaged and analyzed.

Table S1. Results of SDL screens. The “Information” sheet lists the meanings of column headings. The subsequent sheets contain the normalized colony size data for the two SDL screens with wt PTEN along with the pEGH control screen and the C124S mutant screen.

Click here to Download Table S1

Table S2. Reference data set for logistic regression analysis. The table summarizes curated data from the ClinVar, COSMIC and gnomAD databases and assigns a ground truth (1 – pathogenic; 0 – benign) for each variant that has sufficient data available.

Click here to Download Table S2

References

Young, B. P. and Loewen, C. J. R. (2013). Balony: a software package for analysis of data generated by synthetic genetic array experiments. *BMC Bioinformatics* **14**, 354.
